# Analysis of initial stress distribution in palatal bone around the implant in lingual orthodontics for single and double palatal implant systems: a FEM study

**DOI:** 10.1590/2177-6709.27.4.e2120492.oar

**Published:** 2022-09-23

**Authors:** Ashish KUSHWAH, Mukesh KUMAR, Shruti PREMSAGAR, Sonika SHARMA, Sumit KUMAR, Tamada SAILESH

**Affiliations:** 1 Institute of Dental Education & Advance Studies, Department of Orthodontics and Dentofacial Orthopaedics, (Gwalior/MP, India).; 2 Teerthanker Mahaveer Dental College, Department of Orthodontics and Dentofacial Orthopaedics, (Moradabad/UP, India).; 3 ITS Dental College, Department of Orthodontics and Dentofacial Orthopaedics (Greater Noida, UP, India).; 4 Consultant orthodontist (Moradabad/UP, India).; 5 Consultant orthodontist (Vizag, Andhra Pradesh).

**Keywords:** Finite element method, Palatal bone, Palatal implants, Principal stress, Von Mises stress

## Abstract

**Objective:**

To analyze and compare the Von Mises stress and principal stress distribution in palatal bone around the palatal implant in lingual orthodontics (LiO) for single and double palatal implant systems with varying lengths of lever arm.

**Methods:**

Two groups were assessed: single (Group 1) and double (Group 2) palatal implant systems, which were further divided into two subgroups, based on lever arm length, for analyzing stress in the palatal bone around the implant. Hence, two 3D finite element models of bilateral maxillary first premolar extraction cases were constructed in each system. Lingual brackets (0.018-in slot) were positioned at the center of the clinical crown. In both systems, 150g of retraction force was applied, and ANSYS v. 12.1 software was used to analyze and compare stress in the palatal bone around the palatal implant.

**Results:**

In this study, higher stress was observed at the inner threaded interface of cortical bone. Magnitude of Von Mises stress was higher in Group 2 (0.63 MPa and 0.65 MPa) in comparison to Group 1 (0.29 MPa and 0.29 MPa). Similarly, magnitude of principal stress was higher in Group 2, in comparison to Group 1. Higher stress was observed in the apical region of the implant-bone interface of cancellous bone.

**Conclusion:**

This study concluded that the Von Misses stress as well as principal stress in the palatal bone were within the optimal limit in both groups. Finally, it can be concluded that both systems (single and double palatal implant) were safe for the patients in clinical use of 150g of retraction force.

## RESUMO

**Objetivo:**

Analisar e comparar as tensões de Von Mises e a distribuição das tensões principais no osso palatino ao redor de implantes palatinos em Ortodontia Lingual (OL) para sistemas de implantes palatinos unitários ou duplos com comprimentos variados do braço de alavanca.

**Métodos:**

Foram delineados dois grupos para o presente estudo: Grupo 1 - com sistema de implante palatino unitário e Grupo 2 - com sistema de implantes palatinos duplos. Em seguida, os grupos foram divididos em dois subgrupos, com base no comprimento do braço de alavanca, para analisar as tensões no osso palatino ao redor do implante. Para cada sistema, foram construídos dois modelos 3D de elementos finitos (MEF) de casos com extração bilateral de primeiros pré-molares superiores. Braquetes linguais (*slot* 0,018”) foram posicionados no centro das coroas clínicas. Nos dois sistemas, foram aplicados 150g de força de retração nos dentes anteriores, e o *software* ANSYS v. 12.1 foi usado para analisar e comparar as tensões no osso palatino ao redor dos implantes.

**Resultados:**

Foram observados maiores níveis de tensões na parte interna rosqueada no osso cortical. A magnitude das tensões de Von Mises foi maior no Grupo 2 (0,63MPa e 0,65MPa) em comparação ao Grupo 1 (0,29MPa e 0,29MPa). De forma semelhante, foi observada maior magnitude das tensões principais no Grupo 2 do que no Grupo 1. Maiores tensões foram observadas na região apical da interface osso/implante no tecido ósseo esponjoso.

**Conclusão:**

A tensões de Von Mises e as tensões principais no osso palatino ficaram dentro do limite ideal em ambos os grupos. Ambos os sistemas de implantes palatinos (unitário e duplo) foram seguros para o uso clínico em pacientes com força de retração de 150g.

## INTRODUCTION

Rather than the type of appliance used, the success of lingual orthodontics treatment depends on the principles of biomechanics being applied.^1^ The biomechanics involved in the second phase of orthodontic treatment (space closure) is either friction mechanics (*en-masse* retraction/sliding mechanics) or frictionless mechanics (loop mechanics). The success of the orthodontic treatment depends on both structural balance as well as facial esthetics, which is obtained with an optimal anchorage. In the field of orthodontics, implants have attained huge popularity and are being used for orthodontic anchorage.^2^ Dental implants are of different types, and include miniplates, disc-shaped, endosseous, and micro- or miniscrews implants. These implants are considered as successful specially when mechanical stresses are not transmitted to surrounding bone, thus increasing its longevity.^3^

Compared to conventional labial appliances, lingual orthodontics provides greater anchorage stability. In this technique, implants are used to attain intrusive forces and bodily tooth movements, in addition to controlling the anterior loss of torque. The palate is considered as the best location for implant placement. This is due to a good quantity of bone, being easily reached, less prone to inflammation, and safe to work on. The most workable and acceptable area on the palate is the paramedian zone, as it has a low supply of blood vessels and nerves, thus preventing injuries to the underlying tissues.^4^

The most common complication related to implants in orthodontics is noticed to be their fracture. To reduce the fractures, the implant diameter is increased, but this in turn increases the torque, and may cause injury to the underlying structures.^5^ It is not possible to assess intraorally the stress concentration on implants, but this became possible with the advent of an advanced technique called finite element method (FEM), which is a three-dimensional virtual modeling method that makes use of appropriate boundary conditions and load.^6^ To estimate the level of failure, the Von Mises stress is utilized based on the Von Mises yield criterion, which states that material shows yielding when the level of Von Mises stress surpasses the yield strength.^7^This criterion applies to ductile materials, such as metals; while for brittle materials like bone, their maximum principal stress criterion is measured. This criterion states that failure happens when the stress level achieves the level of ultimate tensile or compressive strength.^8^

To achieve the force required for retraction, appropriate implant system and optimal lever arm are necessary. It is crucial to plan the location and line of action of applied force. In the mechanics for lingual retraction, it is necessary to control the torque on anterior teeth by using the first-class lever principle. The position of the implant and the length of the lever arm determines the required line of action of retraction force, in relation to the center of resistance of the anterior segment. The force is adjusted based on the center of resistance of teeth to be moved. In a study by Vanden Bulcke et al.,^9^ it was stated that the center of resistance of the six anterior teeth was located between the central incisors, 7.0 mm apical to the interproximal bone level. It must be considered that the effect of torque is dependent on the lever arm length.

With this background, the present study was planned to analyze the stress distribution in the palatal bone surrounding the implant in lingual orthodontics for single and double palatal implant systems with varying lengths of lever arm, using the finite element method.

## MATERIAL AND METHODS

### STUDY DESIGN

**Figure 1:**
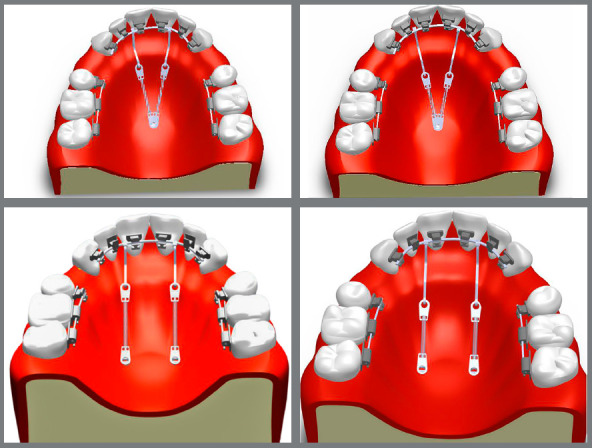
Study models design.

Two groups were created, based on the number of palatal implants used for the *en-masse* retraction of anterior teeth in lingual orthodontics (LiO). In Group 1, single palatal implant (2 mm x 10 mm, SK Surgical) was used at midpalatal raphae between the first and second maxillary molars. In Group 2, two palatal implants (2 mm x 10 mm, SK Surgical) were used (one on each palatal half) at 5 mm away from midpalatal raphae between the first and second maxillary molars. These groups were further divided into two subgroups, based on the length of the lever arm. This lever arm was attached to 0.016 x 0.022-in stainless steel archwire between the central incisor and lateral incisor. Subgroup 1 had a 12-mm long lever arm and Subgroup 2 had a 15-mm long lever arm. The applied amount of force for *en-masse* retraction was 150g on each lever arm (Fig 1).

## METHODOLOGY

Finite element analysis was performed according to the following six steps^10^ ^,^ ^11^: a) construction of the geometric model of the maxillary dentition with its periodontal structures (periodontal ligament, alveolar bone); b) conversion of the geometric models to a finite element model; c) incorporation of the material properties of tooth structure and periodontium; d) defining boundary condition; e) loading configuration; f) translation of results and interpretation.

The CT scan images of maxilla with maxillary dentition were taken in the axial plane and saved as DICOM format. This data was exported to a 3D image processing and editing software (MIMICS v. 8.11, Materialise’s Interactive Medical Image Control System) and, with the help of RapidForm 2004 software, a geometric model was constructed, consisting of only surface data. Lingual brackets (0.018-in slot, Ormco 7^th^ generation), segmented archwire (0.016 x 0.022-in SS), titanium mini-implants (2 mm x 10mm, SK Surgical), NiTi closed coil springs and lever arms (12 mm and 15mm) were virtually modeled using reverse engineering technique. The reverse-engineering process involves measuring an object and then reconstructing it as a 3D model. The physical object can be measured using 3D scanning technologies like laser scanners, structured light digitizers, or industrial CT scanning (computed tomography).

The mesh of the periodontal ligament (PDL) was considered with a uniform thickness of 0.25 mm, according to Coolidge’s study.^12^ Three-dimensional surface to surface sliding contacts with 0.1 coefficient of friction were used between bracket and wire. The contact and friction condition between the archwire and bracket was linear in nature. Models were simulated at a 0.016 x 0.022-in stainless steel archwire stage; therefore, the contact condition between teeth and brackets was closely attached. In Group 1, implant was placed at midpalatal suture (higher position), between first and second molars. In Group 2, implants were placed 5 mm away from midpalatal suture, between first and second molars. A 2 x 10mm implant with the head exposed in the palate provides a straight line of force through lever arm to the teeth.

Geometric models were imported to Hypermesh v. 11.0 software, and all the individual parts - like bone, teeth, periodontal ligament, brackets, wire and mini-implants - were assembled together. By a ‘meshing’ process, Hypermesh v. 11.0 software converted the geometric models into finite element models.

The finite element model is representative of the geometry, in terms of the finite number of elements and nodes. This process is called ‘discretization’. These elements are interconnected at joints, which are called nodes or nodal points, while the corner nodes are called primary external nodes. The additional nodes that occur on the sides of the element are called secondary external nodes. The secondary nodes have fewer displacements than the corner nodes. For maxilla, a 4-noded tetrahedral shape was selected as the finite element, since this element is more suitable for meshing irregular geometries. In Group 1, subgroups 1 and 2 presented number of nodes equal to 86,841 and 86,859, and number of elements equal to 406,925 and 406,948, respectively. In Group 2, subgroups 1 and 2 presented number of nodes equal to 899,496 and 899,512; and number of elements equal to 422,763 and 406,986 respectively.

Material properties of bone, teeth, periodontal ligament, brackets, mini-implants, archwire and NiTi closed coil were incorporated in models (Table 1). The boundary condition of these FEM models needs to be defined so that all movements of the model are restrained, to prevent the model from any type of body motion while the load is acting. For the above mentioned models, the fixed boundary condition was maintained at the base of the maxilla and was constrained in all models.

**Table 1:** Material properties used in the Finite Element Method models.

S. nº.	Materials	Young’s modulus (MPa)	Poisson’s ratio
1.	Hard bone	13700	0.38
2.	Soft bone	1370	0.38
3.	Periodontal ligament (PDL)	0.068	0.49
4.	Teeth	20000	0.30
5.	Titanium implants	11,0000	0.30
6.	SS wire	20,0000	0.30
7.	NiTi closed coil	75,000	0.33
8.	Bracket	21,4000	0.30

The loading configuration was designed to mimic the type of orthodontic tooth movement applied for retraction of the maxillary anterior teeth using NiTi closed coil springs and mini-implants. In all four models, 150g retraction force was applied bilaterally from the mini-implants to the segmented archwire of the anterior segment with lever arms. Finite element models were imported into ANSYS v. 12.1 software for analyzing the displacement and stress distribution.

The following colour coding for stress and displacement was used in the FEM analysis: blue colour shows the minimum stress/displacement, red colour shows the maximum.

## STATISTICAL ANALYSIS

In finite element studies, it is enough to validate the analysis results obtained by the software tools with finite element simulation, instead of experimental readings. Thus, statistical analysis is not required.

## RESULTS

### STRESS CONTOURS IN CORTICAL BONE (IMPLANT REGION)

**Figure 2:**
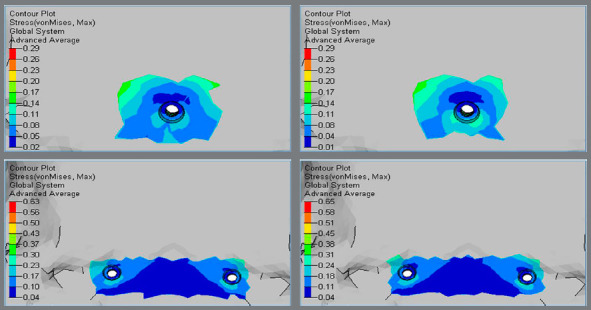
Von Mises stress in the palatal cortical bone around implants.

**Figure 3:**
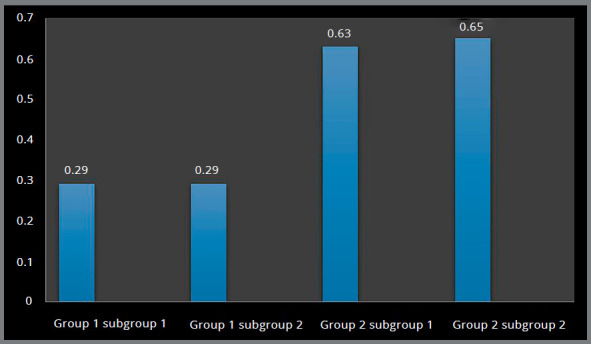
Von Mises stress in the palatal cortical bone around implants.

Higher Von Mises stresses were observed at the inner threaded interface of bone, and magnitude was higher in Group 2 (0.63 MPa and 0.65 MPa) in comparison to Group 1 (0.29 MPa and 0.29 MPa) (Figs 2 and 3, Table 2).

**Table 2:** Von Mises stress and principal stress in cortical bone and cancellous bone for Group 1 and Group 2.

		Group 1		Group 2	
		Subgroup 1	Subgroup 2	Subgroup 1	Subgroup 2
Cortical bone	Von Mises stress (MPa)	0.29	0.29	0.63	0.65
	Principal stress (MPa)	0.29	0.30	0.49	0.46
Cancellous bone	Von Mises stress (MPa)	0.21	0.22	0.26	0.27
	Principal stress (MPa)	0.22	0.22	0.26	0.27

**Figure 4:**
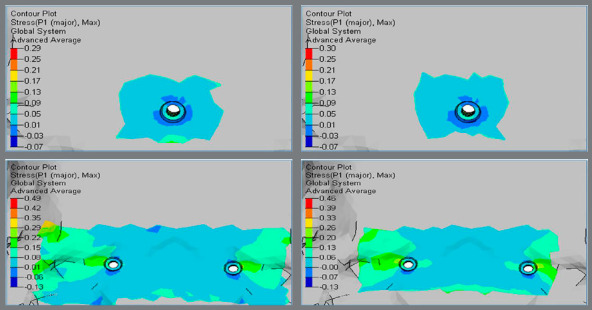
Principal stress in the palatal cortical bone around implants.

**Figure 5:**
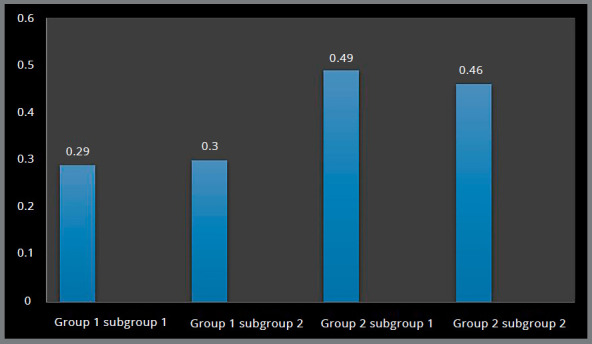
Principal stress in the palatal cortical bone around implants.

Higher principal stresses were observed at the inner threaded interface of bone, and magnitude was higher in Group 2 (0.49 MPa and 0.46 MPa), in comparison to Group 1 (0.29 MPa and 0.30 MPa) (Figs 4 and 5, Table 2).

### STRESS CONTOURS IN CANCELLOUS BONE (IMPLANT REGION)

**Figure 6:**
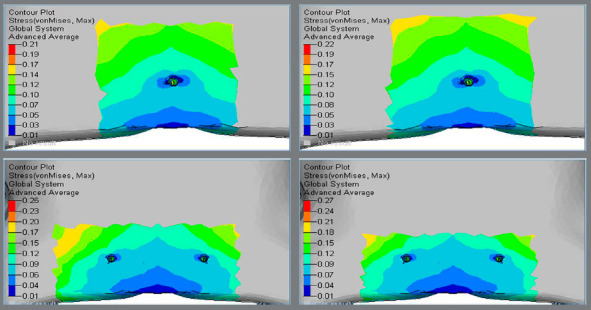
Von Mises stress in the palatal cancellous bone around implants.

**Figure 7:**
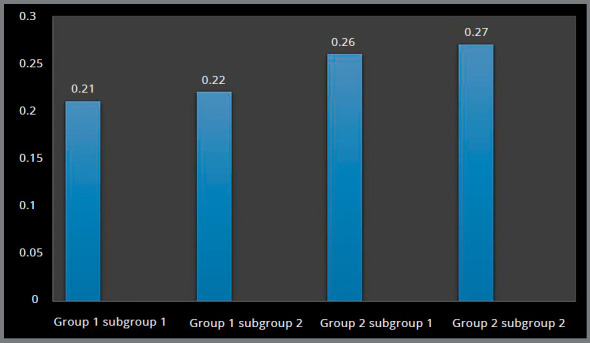
Von Mises stress in the palatal cancellous bone around implants.

Higher Von Mises stresses were observed at the apical region of the implant-bone interface, and magnitude was higher in Group 2 (0.26 MPa and 0.27 MPa) in comparison to Group 1 (0.21 MPa and 0.22 MPa) (Figs 6 and 7, Table 2).

**Figure 8:**
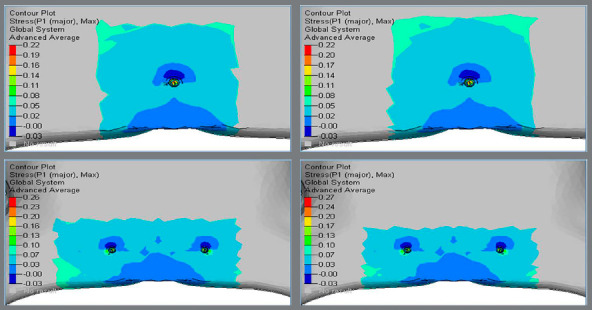
Principal stress in the palatal cancellous bone around implants.

**Figure 9:**
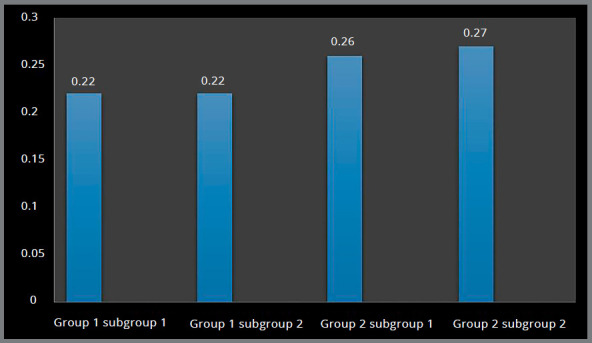
Principal stress in the palatal cancellous bone around implants.

Higher principal stresses were observed at the apical region of the implant-bone interface, and magnitude was higher in Group 2 (0.26 MPa and 0.27 MPa), in comparison to Group 1 (0.22 MPa and 0.22 MPa) (Figs 8 and 9, Table 2).

## DISCUSSION

In the 1970s, Dr. Kurz and Dr. Fujita invented the Lingual Orthodontics system.^13^ It is observed that with lingual orthodontics, the torque generated on the maxillary incisors is difficult to control during the incisor retraction. 

Nowadays, orthodontic mini-implants are being used with good survival and success rates. They are known to provide a stable anchorage in orthodontic treatment, but one should consider various critical issues, to prevent future complications with these mini-implants. One of these critical issues is the site of implant insertion.

Lee et al.^14^advocated the insertion of mini-implants in the palatal suture. They reported that palatal implants were more successful than buccal implants, as the keratinized soft tissue in the palate is lined with a slightly thinner palatal bone. This allows a faster intrusion and patients even report with a better state of general well-being. Thus in the present study, it was also used the midpalatal suture area as the site for implant insertion. 

In a study by Hong et al.^15^, it has been observed that when the lever arm length is modified in relation to mini-implant location, a required retraction force is achieved in relation to the center of resistance for the anterior teeth. This guarded anterior teeth retraction was accomplished without loss of anchorage. Thus, they found that when mini-implant is used with lever arm, it provides an absolute anchorage and control the torque in the anterior segment through the anterior teeth retraction in lingual orthodontic treatment. Thus, the present study was carried to analyze the effect of changing the lever arm length on cortical and cancellous bones during incisor retraction.

It was observed that stress concentration was higher in double implants placed in cortical bone, and also with longer lever arms. Similar to this study, Hong et al.^15^ found that better results were achieved with traction using a shorter lever arm. They also found that a successful lingual treatment result was observed after*en-masse*retraction with the use of a single wire with a smaller length of the arm. However, if the length of the lever arm is greater than 15 mm, it leads to elastic deformation; thus, causing bowing in the anterior transverse region, thus diminishing the translational effects on the incisors.^16^ Similarly, Lim and Hong^17^also found that the double-wire technique is much more successful in maintaining the incisor inclination using a shorter arm. However, in conventional lingual orthodontics, mini-implant anchorage with longer lever arm is used for retraction. This displays a common side effect, which is limited flexibility of the archwire as well as torque loss due to the slot play in the appliance.

The present study allowed to observe that the stresses were more concentrated on cortical than on cancellous bone. But in a study by Liu et al.^18^, it was advocated that for the mini-implant stability, the cancellous bone quality is not a decisive parameter; however, the thickness of the cortex should be at least 1.2 mm.

Moon et al.^19^ also measured the midpalatal bone density in adult subjects, and stated that bone density tends to decrease from anterior to posterior areas, and from middle to lateral areas of the palate.

Despite palatal bone being thickest in the midpalatal suture (MPS) region, it is not an ideal site for anchorage purposes, due to inadequate calcification and interposition of connective tissue, especially in young growing children. Hence, the alternate optimum site is the paramedian region, 3 mm lateral to the MPS.^20^ We opted for 10-mm long mini-implant because the palatal bone thickness^21^ ^,^ ^22^ between first and second molars ranges from 4 to 5 mm, and the palatal mucosa thickness^23^ ranges from 5 to 6 mm.

The present study used FEM (finite element method), which has become an effective method for oral biomechanics research, since the development of digital technology.^24^ ^,^ ^25^ It is one of the most appropriate methods to evaluate the orthodontic movement of teeth.^26^ FEM can evaluate the qualitative and quantitative effects on the alveolar bone, dentition, and periodontal ligament.

### LIMITATIONS OF THE STUDY

In the present study, all the material properties were considered as ideal values, as observed by previous studies. But readings can vary among individuals and with different palatal positions. 

The present study has not considered a few factors that can affect the results, including bone density and thickness.

The present study was elaborated considering the mechanical properties of materials used, so it can be correlated to clinical conditions and obtain the best outcome, for the benefit of patient and clinician.

### CLINICAL IMPLICATION

The palatal bone stresses around palatal implant were compared in single *versus* double palatal implant systems, and it was additionally found that both the groups can work in clinical scenario without failure.

In the case of double implants system, the length of the lever arm did not show any remarkable effect in the palatal bone.

## CONCLUSION

It can be concluded that Von Mises stress of palatal bone decreased as the palatal implant was placed at a higher position (at midpalatal suture) (0.29 MPa and 0.29 MPa) in the palate, but the amount of stress did not exceed the optimum limit. In both groups, the lever arm did not show any remarkable effect in the palatal bone. The highest amount of the principal stress was observed in the threaded interface of palatal bone and palatal implant. All two groups are clinically safe because the amount of Von Mises stress was within the optimum limit.

Finally, it can be concluded that regarding single *versus* double palatal implant system, the double palatal implant system was safe for the patients palatal bone in clinical use of 150g of retraction force.
